# Comparative Genome Analysis of Filamentous Fungi Reveals Gene Family Expansions Associated with Fungal Pathogenesis

**DOI:** 10.1371/journal.pone.0002300

**Published:** 2008-06-04

**Authors:** Darren M. Soanes, Intikhab Alam, Mike Cornell, Han Min Wong, Cornelia Hedeler, Norman W. Paton, Magnus Rattray, Simon J. Hubbard, Stephen G. Oliver, Nicholas J. Talbot

**Affiliations:** 1 School of Biosciences, Geoffrey Pope Building, University of Exeter, Exeter, United Kingdom; 2 School of Computer Science, University of Manchester, Manchester, United Kingdom; 3 Faculty of Life Sciences, Michael Smith Building, University of Manchester, Manchester, United Kingdom; 4 Department of Biochemistry, University of Cambridge, Sanger Building, Cambridge, United Kingdom; Centre for DNA Fingerprinting and Diagnostics, India

## Abstract

Fungi and oomycetes are the causal agents of many of the most serious diseases of plants. Here we report a detailed comparative analysis of the genome sequences of thirty-six species of fungi and oomycetes, including seven plant pathogenic species, that aims to explore the common genetic features associated with plant disease-causing species. The predicted translational products of each genome have been clustered into groups of potential orthologues using Markov Chain Clustering and the data integrated into the *e-Fungi* object-oriented data warehouse (http://www.e-fungi.org.uk/). Analysis of the species distribution of members of these clusters has identified proteins that are specific to filamentous fungal species and a group of proteins found only in plant pathogens. By comparing the gene inventories of filamentous, ascomycetous phytopathogenic and free-living species of fungi, we have identified a set of gene families that appear to have expanded during the evolution of phytopathogens and may therefore serve important roles in plant disease. We have also characterised the predicted set of secreted proteins encoded by each genome and identified a set of protein families which are significantly over-represented in the secretomes of plant pathogenic fungi, including putative effector proteins that might perturb host cell biology during plant infection. The results demonstrate the potential of comparative genome analysis for exploring the evolution of eukaryotic microbial pathogenesis.

## Introduction

Fungi and oomycetes are responsible for many of the world's most devastating plant diseases including late blight disease of potato, caused by the oomycete pathogen *Phytophthora infestans* and rice blast disease caused by the ascomycete fungus *Magnaporthe grisea*, both of which are responsible for very significant harvest losses each year. The enormous diversity of crop diseases caused by these eukaryotic micro-organisms poses a difficult challenge to the development of durable disease control strategies. Identifying common underlying molecular mechanisms necessary for pathogenesis in a wide range of pathogenic species is therefore a major goal of current research. Approximately 100,000 species of fungi have so far been described, but only a very small proportion of these are pathogenic [1]. Phylogenetic studies have, meanwhile, shown that disease-causing pathogens are not necessarily closely-related to each other, and in fact are spread throughout all taxonomic groups of fungi, often showing a close evolutionary relationship to non-pathogenic species [2], [3]. It therefore seems likely that phytopathogenicity has evolved as a trait many times during fungal and oomycete evolution [1] and in some groups may be ancestral to the more recent emergence of saprotrophic species. A significant effort has gone into the identification of pathogenicity determinants– individual genes that are essential for a pathogen to invade a host plant successfully, but which are dispensable for saprophytic growth [4], [5]. However, far from being novel proteins encoded only by the genomes of pathogenic fungi, many of the genes identified so far encode components of conserved signalling pathways that are found in all species of fungi, such as the mitogen activated protein (MAP) kinases [6], adenylate cyclase [7] and G-protein subunits [8]. The MAP kinase pathways, for example, have been studied extensively in the budding yeast *Saccharomyces cerevisiae* and trigger morphological and biochemical changes in response to external stimuli such as starvation stress or hyperosmotic conditions [9]. In pathogenic fungi, components of these pathways have evolved instead to regulate the morphological changes associated with plant infection. For example, appressorium formation in the rice blast fungus *Magnaporthe grisea*, stimulated by hard, hydrophobic surfaces is regulated by a MAP kinase cascade [10]. This pathway deploys novel classes of G-protein coupled receptors not found in the genome of *S. cerevisiae* [11], but the inductive signal is transmitted via a MAP kinase, Pmk1, that is a functional homologue of the yeast Fus3 MAP kinase where it serves a role in pheromone signalling [10]. Similarly, conserved metabolic pathways such as the glyoxylate cycle and amino acid biosynthesis are also important for pathogenesis [12]–[14]. This may in some cases reflect the nutritional environment the pathogen encounters when growing in the host plant tissue, and in others shows the importance of simple metabolites for pathogenic processes, such as the role of glycerol as a compatible solute for generating turgor pressure in the appressorium of *M*. *grisea* [15]. It is undoubtedly the case, however, that identification of such genes has also been a consequence of the manner in which these studies have been carried out, often using yeast as a model organism to test hypotheses concerning the developmental biology and biochemistry of plant pathogenic species.

Other pathogenicity factors identified to date have been shown to be involved in functions associated with host infection, such as plant cell wall degradation, toxin biosynthesis and protection against plant defences [reviewed in 5]. Identification of a pathogenicity factor generally involves making a mutant fungal strain with a non-functioning version of the gene by targeted gene deletion and assaying the ability of the mutant to cause disease. Therefore, most pathogenicity factors identified so far, have been validated in only a small number of genetically tractable pathogenic fungi, such as *M. grisea* and the corn smut *Ustilago maydis* and many of the advances in understanding the developmental biology of plant infection have occurred in these model pathogens [16], [17]. However, there are severe limitations to studying pathogenicity by mutating one gene at a time and working predominantly with a hypothesis-driven, reverse genetics approach. Many virulence-associated processes, for instance, such as the development of infection structures and haustoria, are likely to involve a large number of gene products and so there is likely to be redundancy in gene function. One example of this is cutinase, a type of methyl esterase that hydrolyses the protective cutin layer present on the outside of the plant epidermis. Cutinase was excluded as a pathogencity factor for *M. grisea* on the basis that a mutant strain containing a non-functional cutinase-encoding gene was still able to cause rice blast disease [18]. However, sequencing of the *M. grisea* genome has shown the presence of eight potential cutinase-encoding genes implicated in virulence [19]. Additionally, targeted gene deletion is not feasible in many important pathogens and the normal definition of fungal pathogenicity cannot be applied in the case of obligate biotrophs, such as the powdery mildew fungus *Blumeria graminis*, which cannot be cultured away from living host plants. Therefore, new approaches are needed to identify genes that are vital for the process of pathogenicity. These include high-throughput methods such as microarray analysis, serial analysis of gene expression (SAGE), insertional mutagenesis, proteomics and metabolomics [19], [20] and are dependent on the availability of genome sequence information.

After the initial release of the genome of the budding yeast *S. cerevisiae* in 1996 [21], the number of publicly available sequenced fungal genomes has recently risen very quickly. A large number of fungal genome sequences are now publicly available, including those from several phytopathogenic fungi, including *M. grisea* [22], *Ustilago maydis* [23], *Gibberella zeae* [24] (the causal agent of head blight of wheat and barley), *Stagonospora nodorum* [25] (the causal agent of glume blotch of wheat), the grey mould fungus *Botrytis cinerea* and the white mould fungus *Sclerotinia sclerotiorum* [reviewed in 19]. Comparison of gene inventories of pathogenic and non-pathogenic organisms offers the most direct means of providing new information concerning the mechanisms involved in fungal and oomycete pathogenicity. In this report, we have developed and utilized the *e-Fungi* object-oriented data warehouse [26], which contains data from 36 species of fungi and oomycetes and deploys a range of querying tools to allow interrogation of a significant amount of genome data in unparalleled detail. We report the identification of new gene families that are over represented in the genomes of filamentous ascomycete phytopathogens and define gene sets that are specific to diverse fungal pathogen species. We also report the putatively secreted protein sets which are produced by plant pathogenic fungi and which may play significant roles in plant infection.

## Results

### Identification of orthologous gene sets from fungal and oomcyete genomes

Genome sequences and sets of predicted proteins were analysed from 34 species of fungi and 2 species of oomycete (Table 1). In order to compare such a large number of genomes, an object-oriented data warehouse has been constructed known as *e-Fungi* [26] which integrates genomic data with a variety of functional data and has a powerful set of queries that enables sophisticated, whole-genome comparisons to be performed. To compare genome inventories, the entire set of predicted proteins from the 36 species (348,787 proteins) were clustered using Markov Chain Clustering [27] as described previously [28], [29]. A total of 282,061 predicted proteins were grouped into 23,724 clusters, each cluster representing a group of putative orthologues. The remaining 66,934 sequences were singletons, the products of unique genes. A total of 165 clusters contained proteins from all 36 species used in this study (Table S1). Not surprisingly, they included many proteins involved in basic cellular processes, such as ribosomal proteins, components of transcription, translation and DNA replication apparatus, cytoskeletal proteins, histones, proteins involved in the secretory pathway, protein folding, protein sorting and ubiquitin-mediated proteolysis and enzymes involved in primary metabolism. Only 16 clusters contained proteins that were found in all 34 species of fungi, but which were absent from the two species of oomycete (Table S2). This number of fungal-specific clusters is surprisingly low considering the phylogenetic distance between the oomycetes and fungi [30]. The list however, is consistent with the fundamental differences in biology between fungi and oomycetes and included proteins involved in fungal septation, glycosylation, transcriptional regulation, cell signalling, as well as two amino-acyl tRNA synthetases. The obligate mammalian pathogen *Encephalitozoon cuniculi*, a microsporidian fungus, has a reduced genome that codes only for 1,997 proteins and lacks genes encoding enzymes of many primary metabolic pathways such as the tricarboxylic acid cycle, fatty acid β-oxidation, biosynthetic enzymes of the vast majority of amino acids, fatty acids and nucleotides, as well as components of the respiratory electron transport chain and F_1_-F_0_ ATP synthase. It also lacks mitochondria and peroxisomes [31]. Therefore, we reasoned that the inclusion of this species in the analysis of MCL clusters is likely to result in underestimation of the number of groups of conserved proteins. By discarding *E. cuniculi*, there are 377 clusters that contained proteins from 35 species of fungi and oomycetes (Table S3). This relatively small number of fungal-conserved clusters reflects the large evolutionary distance between members of the fungal kingdom, as well as complex patterns of gene gains and losses during the evolution of fungi. Basidiomycetes and ascomycetes are thought to have diverged nearly 1,000 million years ago [32] and the Saccharomycotina alone are more evolutionarily diverged than the Chordate phylum of the animal kingdom [33]. Since the divergence of Saccharomycotina (hemiascomycetes) and Pezizomycotina (euascomycetes), the genomes of the latter have greatly increased in size, partly due to the appearance of novel genes related to the filamentous lifestyle. Lineage-specific gene losses have also been shown in a number of hemiascomycete species [34]. As well as the groups of proteins mentioned above (Table S1), the fungal-conserved clusters included those containing enzymes from primary metabolic pathways not present in *E. cuniculi*, such as the tricarboxylic acid cycle, amino acid metabolism, fatty acid biosynthesis, cholesterol biosynthesis and nucleotide metabolism, as well as components of the respiratory electron transport chain and F_1_-F_0_ ATP synthase. The conserved protein clusters also include a number of transporters (including mitochondrial transporters), enzymes involved in haem biosynthesis, autophagy-related proteins, those involved in protein targeting to the peroxisome and vacuole and additional groups of proteins involved in signal transduction that are not present in *E. cuniculi* (including those involved in inosine triphosphate and leukotriene metabolism). The analysis also showed there were 105 clusters that contained proteins from 33 species of fungi (excluding *E. cuniculi*), but not from the two species of oomycete (see Table S4). As well as those mentioned previously (Table S2), the group includes a number of clusters of transporters that are conserved in fungi but not found in oomycetes, as well as proteins involved in fungal cell wall synthesis, and lipid metabolism. It may be the case that the genomes of oomycete species do not possess orthologues of the fungal genes in these clusters, or alternatively, the large evolutionary distance between the oomycetes and fungi mean that the corresponding orthologues from each Kingdom cluster separately.

**Table 1:** Fungal species used in this study

Species	Website	Reference (if published)
Aspergillus fumigatus	http://www.sanger.ac.uk/Projects/A_fumigatus/	106
*Aspergillus nidulans*	http://www.broad.mit.edu/annotation/genome/aspergillus_group/MultiHome.html	107
Aspergillus niger	http://genome.jgi-psf.org/Aspni1/Aspni1.home.html	108
*Aspergillus oryzae*	http://www.bio.nite.go.jp/ngac/e/rib40-e.html	109
*Aspergillus terreus*	http://www.broad.mit.edu/annotation/genome/aspergillus_group/MultiHome.html	
*Botrytis cinerea*	http://www.broad.mit.edu/annotation/genome/botrytis_cinerea/Home.html	
*Candida albicans*	http://www.candidagenome.org/	110
*Candida glabrata*	http://cbi.labri.fr/Genolevures/elt/CAGL	33
*Candida lusitaniae*	http://www.broad.mit.edu/annotation/genome/candida_lusitaniae/Home.html	
*Chaetomium globosum*	http://www.broad.mit.edu/annotation/genome/chaetomium_globosum/Home.html	
*Coccidioides immitis*	http://www.broad.mit.edu/annotation/genome/coccidioides_group/MultiHome.html	
*Debaryomyces hansenii*	http://cbi.labri.fr/Genolevures/elt/DEHA	33
*Encephalitozoon cuniculi*	http://www.cns.fr/externe/English/Projets/Projet_AD/AD.html	31
Eremothecium gossypii	http://agd.vital-it.ch/info/data/download.html	111
Gibberella zeae	http://www.broad.mit.edu/annotation/genome/fusarium_graminearum/Home.html	24
*Kluyveromyces lactis*	http://cbi.labri.fr/Genolevures/elt/KLLA	33
*Kluyveromyces waltii*	http://www.nature.com/nature/journal/v428/n6983/extref/S2_ORFs/predicted_proteins.fasta	
Magnaporthe grisea	http://www.broad.mit.edu/annotation/genome/magnaporthe_grisea/Home.html	22
*Neurospora crassa*	http://www.broad.mit.edu/annotation/genome/neurospora/Home.html	112
*Phanerochaete chrysosporium*	http://genome.jgi-psf.org/Phchr1/Phchr1.home.html	113
*Phytophthora ramorum*	http://genome.jgi-psf.org/Phyra1_1/Phyra1_1.home.html	114
*Phytophthora sojae*	http://genome.jgi-psf.org/Physo1_1/Physo1_1.home.html	114
*Rhizopus oryzae*	http://www.broad.mit.edu/annotation/genome/rhizopus_oryzae/Home.html	
*Saccharomyces bayanus*	http://www.broad.mit.edu/annotation/fungi/comp_yeasts/	115
*Saccharomyces castellii*	ftp://genome-ftp.stanford.edu/pub/yeast/data_download/sequence/fungal_genomes/S_castellii/WashU/orf_protein/orf_trans.fasta.gz	
*Saccharomyces cerevisiae*	http://www.yeastgenome.org/	21
*Saccharomyces kluyveri*	http://genome.wustl.edu/genome.cgi?GENOME=Saccharomyces%20kluyveri	
*Saccharomyces kudriavzevii*	ftp://genome-ftp.stanford.edu/pub/yeast/data_download/sequence/fungal_genomes/S_kudriavzevii/WashU/orf_protein/orf_trans.fasta.gz	
*Saccharomyces mikatae*	http://www.broad.mit.edu/annotation/fungi/comp_yeasts/	115
*Saccharomyces paradoxus*	http://www.broad.mit.edu/annotation/fungi/comp_yeasts/	115
*Schizosaccharomyces pombe*	http://www.sanger.ac.uk/Projects/S_pombe/	116
Sclerotinia sclerotiorum	http://www.broad.mit.edu/annotation/genome/sclerotinia_sclerotiorum/Home.html	
Stagonospora nodorum	http://www.broad.mit.edu/annotation/genome/stagonospora_nodorum/Home.html	25
*Trichoderma reesei*	http://genome.jgi-psf.org/Trire2/Trire2.home.html	
Ustilago maydis	http://www.broad.mit.edu/annotation/genome/ustilago_maydis/Home.html	23
*Yarrowia lipolytica*	http://cbi.labri.fr/Genolevures/elt/YALI	33

### Comparative analysis of yeasts and filamentous fungi

One striking difference in the morphology of species of fungi is between those that have a filamentous, multi-cellular growth habit and those that grow as single yeast cells. There is some overlap between these two groups; because some fungi are dimorphic or even pleiomorphic, switching between different growth forms depending on environmental conditions or the stage of their life cycle. For example, the corn-smut fungus *Ustilago maydis* can exist saprophytically as haploid yeast-like cells, but needs to form a dikaryotic filamentous growth form in order to infect the host plant [23]. Generally the genomes of the filamentous fungi contain more protein-encoding genes (9,000–17,000) than those from unicellular yeasts (5,000–7,000), perhaps reflecting their greater morphological complexity and secondary metabolic capacity. *U. maydis*, however, has 6,522 protein encoding genes, perhaps reflecting its lack of extensive secondary metabolic pathways and its potential usefulness in defining the minimal gene sets associated with biotrophic growth [23]. The increase in proteome size in filamentous ascomycetes may be due to the expansion of certain gene families or the presence of novel genes that are essential for the filamentous lifestyle.

For the purposes of this study, the filamentous fungi were defined as the filamentous ascomycetes (subphylum Pezizomycotina), basidiomycetes and zygomycetes and the unicellular fungi were defined as the budding yeasts (order Saccharomycetales), the archiascomycete *Schizosaccharomyces pombe* and the microsporidian fungus *Encephalitozoon cuniculi*. A total of 37 MCL clusters contained proteins from all species of filamentous fungi, but no species of unicellular fungi (Table 2). Interestingly, eight of these clusters also contained proteins from both species of oomycete represented in *e-Fungi*. The filamentous-fungal specific clusters included a number of proteins that are involved in cytoskeletal rearrangements (dedicator of cytokinesis protein, integrin beta-1-binding protein, dynactin p62 family, dynein light intermediate chain 2), it seems likely that these are required for the complex morphological changes that filamentous fungi undergo during their lifecycle and the production of differentiated cells, such as spores, fruiting bodies and infection structures. The results also suggest that filamentous fungal species make a greater use of lipids as signalling molecules than yeast species. For example, the occurrence of filamentous fungal-specific clusters representing two groups of lysophospholipases, as well as ceramidases that are involved in sphingolipid signalling [35] and linoleate diol synthases that can catalyse the formation of leukotrienes [36]. Interestingly, one of the products of linoleate diol synthase has been shown to be a sporulation hormone in *Aspergillus nidulans* [37]. There is also a cluster that represents homologues of a novel human gene (LRP16) that acts downstream of a steroid receptor and promotes cell proliferation [38]. Two clusters of filamentous fungal-specific proteins represent enzymes involved in molypterin biosynthesis (MCL2420, MCL2581). Molypterin is a molybdenum-containing co-factor for nitrate reductase, an enzyme that is known to be absent from the species of yeast used in this study [39]. Both these clusters are also found in oomycetes. There are other clusters representing proteins important for activities specific to filamentous fungi, such as homologues of Pro11 (striatin) which regulates fruiting body formation in *Sordaria macrospora* [40], the vegetatible incompatibility protein HET-E-1, which prevents the formation of heterokaryons between incompatible fungal strains in *Podospora anserina* [41], anucleate primary sterigmata protein A from *Aspergillus nidulans*, which is essential for nuclear migration and conidiophore development [42] and cytochrome P450 and polyketide synthase-encoding genes, both of which are involved in a number of secondary metabolic pathways including toxin biosynthesis [43].

**Table 2:** A list of MCL clusters that are conserved in and specific to filamentous fungi

Cluster ID^1^	Predicted function of members of cluster^2^
MCL94	O-methylsterigmatocystin oxidoreductase (cytochrome P450) (O13345)
MCL147	polyketide synthase (P37693)
MCL924	linoleate diol synthase (Q9UUS2)
MCL1613	acetoacetyl-coenzyme A synthetase (Q9Z3R3)
**MCL1912**	neutral/alkaline non-lysosomal ceramidase (PF04734)
**MCL2061**	homogentisate 1,2-dioxygenase (Q00667)
**MCL2420**	molybdenum cofactor biosynthesis protein (Q9NZB8)
**MCL2503**	metal tolerance protein (Q9M2P2)
**MCL2515**	serine protease (Q9QXE5)
**MCL2581**	gephyrin (Q9NQX3)
MCL2664	similar to bacterial membrane protein (Q8YSU5)
MCL2812	vegetatible incompatibility protein HET-E-1 (Q00808)
MCL2938	2-nitropropane dioxygenase (PF03060)
MCL3026	saccharopine dehydrogenase (Q8R127)
**MCL3203**	lysophospholipase (O88202)
MCL3466	cAMP-regulated guanine nucleotide exchange factor II (Q9EQZ6)
MCL3490	cytosolic phospholipase A2 (P50392)
MCL3518	similar to human LRP16 (Q9BQ69)
**MCL3545**	COP9 signalosome complex subunit 6 (O88545)
MCL3546	anucleate primary sterigmata protein A (Q00083)
MCL3547	dynein light intermediate chain 2, cytosolic (O43237)
MCL3573	3-oxoacyl-[acyl-carrier-protein] reductase (Q9X248)
MCL3665	dedicator of cytokinesis protein 1 (Q14185)
MCL3670	ketosamine-3-kinase (Q8K274)
MCL3770	unknown
MCL3945	integrin beta-1 binding protein 2 (Q9R000)
MCL4010	dynactin p62 family (PF05502)
MCL4033	citrate lyase beta chain (O53078)
MCL4036	peroxisomal hydratase-dehydrogenase-epimerase (multifunctional beta-oxidation protein) (Q01373)
MCL4037	striatin Pro11 (Q70M86)
MCL4054	histone-lysine N-methyltransferase (Q04089)
MCL4055	unknown
MCL4057	protein of unknown function (PF06884)
MCL4058	UV radiation resistance-associated gene protein (Q9P2Y5)
MCL4062	intramembrane protease (P49049)
MCL4068	unknown
MCL4082	mitochondrial protein cyt-4 (P47950)

1 Cluster IDs highlighted in bold type are also found in both species of oomycetes.

2 Predicted function based on best hit against Swiss-Prot protein database (blastp–e-value < = 10^−20^) or Pfam motifs (if no Swiss-Prot hit found). Accession number of top Swiss-Prot hit or Pfam motif is shown in brackets.

### Pathogenicity-associated gene functions in fungi

As the selected set of fungi includes both saprotrophic and pathogenic species, this allows us to compare the gene inventories of phytopathogenic and closely related non-pathogenic fungi to look for genes that are unique to phytopathogens. Analysis of MCL clusters showed that there were no clusters that contained proteins from all species of fungal phytopathogen in *e-Fungi* (namely *B. cinerea*, *Eremothecium gossypii*, *G. zeae*, *M. grisea*, *S. sclerotiorum*, *S. nodorum* and *U. maydis*) but did not contain proteins from non-pathogenic species. There were, however, four clusters that were exclusive to filamentous ascomycete phytopathogens (namely *B. cinerea*, *G. zeae*, *M. grisea*, *S. sclerotiorum*, *S. nodorum* as shown in Table 3). Significantly, none of the members of these clusters had homology to any known proteins or contained motifs from the Pfam database [44], so we were unable to predict their function, although two of the clusters (MCL4854 and MCL8229) consisted entirely of proteins that were predicted to be secreted. Taken together, the observations indicate that a battery of completely novel secreted proteins may be associated with ascomycete fungal pathogens.

**Table 3:** Ascomycete phytopathogen-specific MCL clusters.

Cluster ID	*B. cinerea*	*G. zeae*	*M. grisea*	*S. sclerotiorum*	*S. nodorum*
MCL4854	1	1	6	6	1
MCL8229	1	1	2	1	2
MCL9641	1	1	1	1	1
MCL9651	1	1	1	1	1

MCL clusters containing proteins in all five species of ascomycete pathogen, but no other fungal species. Table shows number of proteins from each species of ascomycete phytopathogen in each MCL cluster.

Pathogenicity factors have been defined as genes that are essential for successful completion of the pathogen lifecycle but dispensable for saprophytic growth [4]. This is an experimental definition based on whether null mutations of a given gene reduce the virulence of the pathogen on its host. We wished to ascertain whether homologues of previously characterised and experimentally-validated pathogenicity factors were limited to the genomes of pathogenic species. A search was therefore made for pathogenicity factors that have been identified experimentally for the species of phytopathogens represented in *e-Fungi* using PHI-base, the plant-host interaction database [45]. The matching locus was identified for each pathogenicity factor in the corresponding genome sequence by comparing a published protein sequence with sets of predicted proteins for each genome using BLASTP. This produced a list of 105 pathogenicity factors, although corresponding loci could not be found in genome sequences for all the published genes (see Table S5). MCL clusters containing these proteins were identified (76 unique clusters) and the species distribution of members of these clusters analysed. In total, 29 of the MCL clusters contained pathogenicity factors with members from at least 34 of the 36 species represented in *e-Fungi* (Table 4). Not surprisingly, many of these clusters contain conserved components of signalling pathways such as protein kinases, adenylate cyclases, G-proteins and cell cycle regulators. Cellular morphogenesis is known to be important for infection of the host plant by many phytopathogens, for example, in appressorium formation in *Magnaporthe grisea* [46] or the switch in the growth form of *Ustilago maydis* from yeast-like growth to filamentous invasive growth [47]. Links between successful plant infection and cell cycle control have also been demonstrated [48]. It seems likely that conserved signalling pathways that control activities, such as mating and morphogenesis in all fungi, have evolved to control processes essential for pathogencity in phytopathogens. Other conserved pathogenicity factors encode enzymes of metabolic pathways that are present in nearly all fungi, but seem to be important for the life cycle of particular pathogenic species, for example, enzymes involved in beta-oxidation of fatty acids, the glyoxylate shunt, amino acid metabolism and the utilisation of stored sugars. When considered together, this may indicate that nutritional conditions which fungi encounter when invading host plant tissue require mobilisation of stored lipids prior to nutrition being extracted from the host plant. Seventeen of the MCL clusters containing pathogenicity factors were specific to filamentous ascomycetes (Table 5). These include a number of enzymes involved in secondary metabolism, such as those involved in the synthesis of the fungal toxin trichothecene in *G. zeae* [43] and those involved in melanin biosynthesis [49], as well as structural proteins, some of which are components of differentiated cell types not seen in yeasts, for example, hydrophobins which are components of aerial structures such as fruiting bodies [50] but are also involved in pathogenicity [16]. There also seems to be a number of filamentous ascomycete specific receptor proteins (transducin beta-subunit, G-protein coupled receptor, tetraspanins) that have evolved in pathogens to be used in sensing environmental cues that are essential for successful infection of the host [51]. The Woronin body is a structure found only in filamentous ascomycetes, and has been shown to be essential for pathogenicity in *M. grisea* [52]. A major constituent of the woronin body, encoded by MVP1, is a pathogenicity factor for *M. grisea*, but also has homologues in nearly all species of filamentous ascomycetes. Two proteins that were initially discovered as being highly expressed in the appressoria of *M. grisea* and essential for pathogenicity (Mas1 and Mas3) [53] also have homologues in a number of species of filamentous fungi (Table 5). Thus, many innovations that have allowed filamentous ascomycetes to have a more complex morphology than unicellular yeasts have also evolved to be essential for plant infection by phytopathogenic species. Interestingly, none of the MCL clusters containing known pathogenicity factors contained members only from phytopathogenic fungi, apart from those that were restricted to just one species. These are therefore likely to represent highly-specialised proteins that have evolved for the specific lifecycle of just one species of phytopathogen, for example the Pwl proteins involved in determining host range of different strains of *M. grisea* [54]. Two of the proteins specific to *M. grisea*, the metallothionein Mmt1 [55] and the hydrophobin Mpg1 [56] are small polypeptides and are members of highly divergent gene families, other members of which do not cluster together using BLASTP.

**Table 4:** MCL clusters containing known pathogenicity factors that have members in at least 34 out of the 36 fungal and oomycete genomes found in e-Fungi.

Cluster ID	Pathogenicity factor^1^	Function	Number of species
MCL11	MGG_06368.5 (CPKA), UM04456.1 (ADR1), UM04956.1 (UKC1), UM03315.1 (UKB1)	cAMP-dependent protein kinase catalytic subunit	36
MCL1121	SNU09357.1 (ALS1)	delta-aminolevulinic acid synthase	35
MCL120	UM01643.1 (RAS2)	guanyl nucleotide exchange factor	35
MCL122	MGG_03860.5 (TPS1)	trehalose-6-phosphate synthase subunit 1	36
MCL1224	MGG_05201.5 (MGB1)	heterotrimeric G-protein beta subunit	34
MCL1495	FG10825.1 (MSY1)	methionine synthase	34
MCL150	BC1G_03430.1 (PIC5)	FKBP-type peptidyl-prolyl cis-trans isomerase	35
MCL1545	MGG_07528.5 (PTH3)	imidazoleglycerol-phosphate dehydratase	34
MCL157	MGG_12855.5 (MST11), UM04258.1 (KPP4)	MAP kinase kinase kinase	35
MCL175	UM02588.1 (CLB2), UM04791.1 (CLN1)	cyclin	35
MCL179	MGG_00800.5 (MST7), UM01514.1 (FUZ7)	MAP kinase kinase	34
MCL193	BC1G_01681.1 (BCG1), SNU10086.1 (GNA1), MGG_00365.5 (MAGB), UM04474.1 (GPA3)	G alpha protein subunit	35
MCL196	MGG_01721.5 (PTH2)	carnitine acetyl transferase	34
MCL24	UM04218.1 (KIN2)	kinesin motor protein	36
MCL244	FG01932.1 (CBL1)	cystathionine beta-lyase	35
MCL248	UM01516.1 (SQL2)	guanyl nucleotide exchange factor	34
MCL295	UM03917.1 (CRU1)	cell cycle regulatory protein	36
MCL42	MGG_00529.5 (PEX6)	peroxin, peroxisome biogenesis	36
MCL421	MGG_06148.5 (MFP1)	multifunctional beta-oxidation enzyme	34
MCL446	MGG_04895.5 (ICL1)	isocitrate lyase	35
MCL46	BC1G_13966.1 (BMP1), FG10313.1 (MGV1), FG06385.1 (MAP1), MGG_04943.5 (MPS1), MGG_09565.5 (PMK1), SNU03299.1 (MAK2), UM03305.1 (KPP2), UM02331.1 (KPP6), UM03305.1 (UBC3)	MAP kinase	36
MCL49	BC1G_01740.1 (BCP1), MGG_10447.5 (CYP1)	cyclophillin	36
MCL54	MGG_06320.5 (CHM1), UM04583.1 (SMU1), UM02406.1 (CLA4)	PAK kinase	35
MCL618	MGG_07335.5 (SUM1), UM06450.1 (UBC1)	cAMP-dependent protein kinase regulatory subunit	35
MCL726	SNU03643.1 (ODC)	ornithine decarboxylase	34
MCL761	SNU07548.1 (MLS1)	malate synthase	34
MCL892	UM04405.1 (GAS1)	alpha-glucosidase	34
MCL9	BC1G_04420.1 (BcatrB), MGG_13624.5 (ABC1)	ABC transporter	35
MCL95	MGG_00111.5 (PDE1), MGG_02767.5 (APT2)	P-type ATPase, aminophospholipid translocase	35

1 Locus ID from the fungal genome projects, first two letters of ID denotes the species, BC = *Botrytis cinerea*, FG = *Fusarium graminearum* (*Gibberella zeae*), MG = *Magnaporthe grisea*, SN = *Stagonospora nodorum*, UM = *Ustilago maydis*. Names of genes encoding pathogenicity factors are enclosed in brackets.

**Table 5:** MCL clusters containing known pathogenicity factors that have members only in the genomes of filamentous ascomycetes

Cluster ID	Pathogenicity factor^1^	Function	Number of species
MCL11972	FG03537.1 (TRI5)	trichodiene synthase	3
MCL14401	FG03536.1 (TRI6)	transcription factor, trichothecene biosynthesis pathway	2
MCL18766	MGG_04301.5 (PWL1/2), MGG_13863.5 (PWL1/2)	host species-specificity protein	1
MCL2795	FG01555.1 (ZIF1)	b-ZIP transcription factor	13
MCL29	BC1G_13298.1 (BTP1), MGG_05871.5 (PTH11)	G-protein coupled receptor	13
MCL4777	MGG_02696.5 (MVP1)	vacuolar ATPase, woronin body protein	12
MCL48738	FG03543.1 (TRI14)	trichothecene biosynthesis gene	1
MCL52178	MGG_06873.5 (ORP1)	essential for penetration of host leaves	1
MCL52784	MGG_09730.5 (MMT1)	metallothionein	1
MCL52927	MGG_10315.5 (MPG1)	class I hydrophobin	1
MCL6180	MGG_04202.5 (MAS1)	highly expressed in appressoria	9
MCL6560	MGG_05059.5 (RSY)	scytalone dehydratase	9
MCL7081	MGG_01173.5 (MHP1)	class II hydrophobin	7
MCL7423	BC1G_09439.1 9 (BcPLS1)	tetraspanin	9
MCL8295	FG00332.1 (TBL1)	transducin beta-subunit	7
MCL8340	MGG_12337.5 (MAS3)	highly expressed in appressoria	6
MCL8912	MGG_00527.5 (EMP1)	extracellular matrix protein	6

1 Locus ID from the fungal genome projects, first two letters of ID denotes the species, BC = *Botrytis cinerea*, FG = *Fusarium graminearum* (*Gibberella zeae*), MG = *Magnaporthe grisea*. Names of genes encoding pathogenicity factors are enclosed in brackets.

### Comparative analysis of plant-pathogenic and saprotrophic filamentous ascomycetes

**Figure 1:**
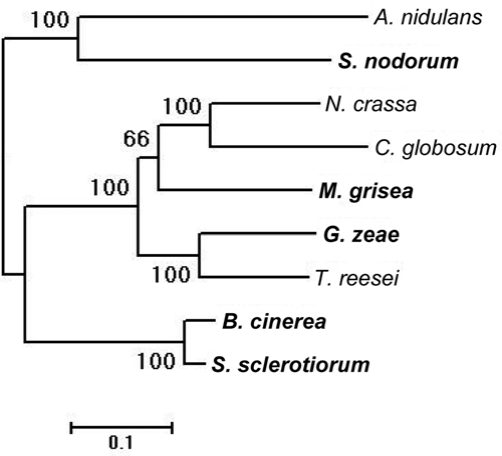
Species tree of filamentous ascomycetes used in this study based on concatenated sequences from 60 universal fungal protein families. Support values shown for each branch (based on 100 bootstraps). Phytopathogenic species are highlighted in bold type. A more detailed methodology has been described previously [26].

Based on the analysis reported, it is likely that in general there are a large number of differences in gene inventories between filamentous and yeast-like fungi. Therefore, in order to compare the genomes of phytopathogens and saprotrophs, we focused on filamentous ascomycetes in order to resolve in greater detail the distinct differences in gene sets between these two ecologically separate groups of fungi. In this way differences due to phylogeny between the species would be minimised. We compared the gene inventories of the phytopathogens *B. cinerea*, *G. zeae*, *M. grisea*, *S. sclerotiorum*, *S. nodorum* with the non-pathogens *Aspergillus nidulans*, *Chaetomium globosum*, *Neurospora crassa* and *Trichoderma reesei*. Phylogenetic analysis suggests that the phytopathogenic species do not form a separate clade from the pathogenic species (Figure 1), [3] and we assumed that differences in gene inventory should therefore reflect lifestyle rather than evolutionary distance. In order for such a comparison to be considered valid, the completeness and quality of the fungal genome sequences used should, however, also be comparable. Table S6 summarises the available data about genome sequence coverage, genome size and the number of predicted proteins for each species. This shows that the genome coverage is greater than 5x and the number of predicted proteins in the range of 10,000–16,000 for all genomes used, suggesting a high level of equivalence between species with regard to sequence quality. From our work it seems unlikely that there are pathogenicity factors conserved in, and specific to, all species of phytopathogen. It may, for instance, be the case that differences in the gene inventories are due to the expansion of certain gene families in the genomes of phytopathogenic species associated with functions necessary for pathogenesis. To define protein families, we used the Pfam database which contains protein family models based on Hidden Markov Models [44], [57]. Sets of predicted proteins for each fungal species in *e-Fungi* were analysed for the occurrence of Pfam motifs and the number of proteins containing each domain across fungal species ascertained. The sets of predicted protein sequences used in this study have been automatically predicted as part of each individual genome project and are likely to contain a number of artefactual sequences. The use of Pfam motifs to define gene families in this study reduces the likelihood of such sequences affecting the data, since Pfam motifs are based on multiple sequence alignments of well-studied proteins.

A small number of Pfam motifs were not found in the proteomes of the filamentous ascomycete non-pathogens, but were found in the proteomes of at least three species of filamentous ascomycete phytopathogens (Table 6). These include the *Cas1p-like* motif (PF07779), found in 4 species of phytopathogen, including five copies in *G. zeae*, and the *Yeast cell wall synthesis protein KRE9/KNH1* motif (PF05390), which was found in three species of phytopathogen. Cas1p is a membrane protein necessary for the O-acetylation of the capsular polysaccharide of the basidiomycete animal pathogen *Cryptococcus neoformans* [58]. KRE9 and KNH1 are involved in the synthesis of cell surface polysaccharides in *S. cerevisiae* [59]. Taken together this suggests that synthesis of cell surface polysaccharides is important for phytopathogens, perhaps helping to shroud the fungus from plant defences. The function of the *YDG/SRA domain* motif (PF02182) is unknown, but is found in a novel mouse cell proliferation protein Np95, in which the domain is important both for the interaction with histones and for chromatin binding *in vivo* [60]. As well as domains of unknown function, the list of phytopathogen-specific Pfam motifs includes *Allophanate hydrolase* (PF02682) which is found in an enzyme involved in the ATP-dependent urea degradation pathway [61], a peptidase motif, an *opioid growth receptor* motif (PF04664) and *Mnd1* (PF03962), which is involved in recombination and meiotic nuclear division [62].

**Table 6:** Pfam motifs that are found in the proteomes from at least three species of phytopathogen, but in no species of filamentous ascomycete non-pathogen. Table shows the number of predicted proteins that contain each Pfam motif.

Pfam accession	Pfam description	*B. cinerea*	*G. zeae*	*M. grisea*	*S. sclerotiorum*	*S. nodorum*
PF07779	Cas1p-like protein	1	5	1	1	0
PF02182	YDG/SRA domain	1	0	1	0	1
PF02682	Allophanate hydrolase subunit 1	0	1	1	0	1
PF03577	Peptidase family C69	0	1	1	0	1
PF03962	Mnd1 family	1	0	0	1	1
PF04664	Opioid growth factor receptor (OGFr) conserved region	1	0	0	1	1
PF05390	Yeast cell wall synthesis protein KRE9/KNH1	1	0	0	1	1
PF05899	Protein of unknown function (DUF861)	1	3	0	1	0
PF06916	Protein of unknown function (DUF1279)	1	0	1	1	0
PF06993	Protein of unknown function (DUF1304)	0	1	1	0	1

To detect potential gene family expansion, we decided to identify Pfam motifs that were present in both phytopathogenic and non-pathogenic species of filamentous ascomycetes, but that were more common in the genomes of the former. The Pfam motifs were ranked on the ratio of the mean number of proteins containing each motif in phytopathogens, when compared to non-pathogens (Table 7). The tables only show ratios of greater than or equal to 2.5. Pfam motifs that were more common in the proteomes of pathogens, include some found in enzymes involved in secondary metabolic pathways. These include novel enzymes that have only previously been studied in non-fungal species, such as the chalcone synthases; type III polyketide synthases involved in the biosynthesis of flavonoids in plants [63] and lipoxygenases; components of metabolic pathways resulting in the synthesis of physiologically-active compounds such as eicosanoids in mammals [64] and jasmonic acid in plants [65] as well as antibiotic synthesis monooxygenases. It seems likely that secondary metabolism is essential in phytopathogenic species for the synthesis of mycotoxins, antibiotics, siderophores and pigments [66], but it may also offer fungal pathogens a distinct alternative means of perturbing host metabolism, cell signalling or plant defence, in contrast to bacterial pathogens that rely on protein secretion to achieve this. There also seems to be number of protease and peptidase domains that are more common in the genomes of phytopathogens as well as domains from two classes of cell-wall degrading enzymes: namely *cutinase* (PF01083) and *Glycosyl hydrolase family 53* (PF07745) which is found in arabinogalactan endo-1,4-beta-galactosidases that hydrolyze the galactan side chains that form part of the complex carbohydrate structure of pectin [67]. Two other domains found in enzymes involved in pectin degradation, *pectinesterase* (PF01095) and *Glycosyl hydrolases family 28* (PF00295) are both more than twice as common in the genomes of phytopathogens than saprotrophs. In contrast, domains found in cellulases have fairly equal distribution between the proteomes of phytopathogens and non-pathogens (data not shown). Therefore, for phytopathogens the most essential enzymes for pathogenesis may well be those that allow the fungus to penetrate the protective cutin layer of the plant epidermis and disrupt the pectin matrix of the plant cell wall in which cellulose fibrils are embedded. Pectin-degrading enzymes have already been shown to be pathogenicity factors in a number of fungi [68]. NPP1 motifs are characteristic of a group of proteins called NLPs (Nep1-like proteins) that trigger defence responses, necrosis and cell death in plants and may act as virulence factors [69]. The NLPs are more common in the genomes of phytopathogenic, when compared to non-pathogenic ascomycetes, but are even more numerous in the proteomes of the oomycetes (64 proteins in *Phytophthora ramorum* and 75 in *Phytophthora sojae*). Proteins containing the *Chitin recognition protein* domain (PF00187) are also very common in the proteomes of phytopathogens (18 in *M. grisea* and 16 in *S. nodorum*). A role for chitin-binding proteins has been proposed in protecting the fungal cell wall from chitinases produced by host plants [70]. There are also two other Pfam motifs, which are more common in the proteomes of phytopathogens, that are found in enzymes involved in the catabolism of toxic compounds, namely *arylesterase* (PF01731) and *EthD protein* (PF07110) which breakdown organophosphorus esters [71] and ethyl tert-butyl ether [72], respectively.

**Table 7:** Pfam motifs that are at least twice as common in the proteomes of filamentous ascomycete phytopathogens, compared to filamentous ascomycete non-pathogens.

Accession	Pfam description	B. cin	G. zea	M. gri	S. scl	S. nod	A. nid	C. glo	N. cra	T. ree	path^1^	non-path^2^	Ratio^3^
PF00195	Chalcone and stilbene synthases, N-terminal domain	1	1	2	1	2	0	0	1	0	1.4	0.25	5.6
PF01731	Arylesterase	1	3	0	2	1	0	0	0	1	1.4	0.25	5.6
PF07110	EthD protein	1	2	0	2	2	0	0	0	1	1.4	0.25	5.6
PF03935	Beta-glucan synthesis-associated protein (SKN1)	2	0	0	2	2	1	0	0	0	1.2	0.25	4.8
PF00024	PAN domain	2	12	2	1	5	0	0	2	2	4.4	1	4.4
PF02128	Fungalysin metallopeptidase (M36)	0	1	2	0	2	0	0	0	1	1	0.25	4.0
PF02705	K+ potassium transporter	1	1	1	1	1	0	0	1	0	1	0.25	4.0
PF07504	Fungalysin/Thermolysin Propeptide Motif	0	1	2	0	2	0	0	0	1	1	0.25	4.0
PF00754	F5/8 type C domain	2	4	1	1	1	0	0	2	0	1.8	0.5	3.6
PF03992	Antibiotic biosynthesis monooxygenase	3	1	1	2	2	1	0	1	0	1.8	0.5	3.6
PF03572	Peptidase family S41	2	1	3	1	6	0	0	2	1	2.6	0.75	3.5
PF00209	Sodium:neurotransmitter symporter family	0	3	3	0	2	1	0	0	1	1.6	0.5	3.2
PF00659	POLO box duplicated region	1	0	1	1	1	0	0	1	0	0.8	0.25	3.2
PF01400	Astacin (Peptidase family M12A)	0	2	0	0	2	0	0	0	1	0.8	0.25	3.2
PF02018	Carbohydrate binding domain	0	2	0	1	1	0	1	0	0	0.8	0.25	3.2
PF02116	Fungal pheromone mating factor STE2 GPCR	1	0	1	1	1	1	0	0	0	0.8	0.25	3.2
PF02244	Carboxypeptidase activation peptide	1	0	1	1	1	0	0	0	1	0.8	0.25	3.2
PF03928	Domain of unknown function (DUF336)	1	1	1	1	4	1	0	0	1	1.6	0.5	3.2
PF05051	Cytochrome C oxidase copper chaperone (COX17)	1	0	1	1	1	1	0	0	0	0.8	0.25	3.2
PF05493	ATP synthase subunit H	1	0	1	1	1	0	1	0	0	0.8	0.25	3.2
PF05631	Protein of unknown function (DUF791)	1	0	1	1	1	0	0	0	1	0.8	0.25	3.2
PF05783	Dynein light intermediate chain (DLIC)	1	0	1	1	1	1	0	0	0	0.8	0.25	3.2
PF01083	Cutinase	11	12	17	8	11	4	5	3	4	11.8	4	3.0
PF00187	Chitin recognition protein	6	8	18	8	16	8	7	0	1	11.2	4	2.8
PF00305	Lipoxygenase	3	1	1	2	0	0	1	1	0	1.4	0.5	2.8
PF00314	Thaumatin family	2	1	1	2	1	0	0	1	1	1.4	0.5	2.8
PF02797	Chalcone and stilbene synthases, C-terminal domain	1	1	2	1	2	0	1	1	0	1.4	0.5	2.8
PF05630	Necrosis inducing protein (NPP1)	2	4	4	2	2	2	1	1	0	2.8	1	2.8
PF07745	Glycosyl hydrolase family 53	2	1	1	2	1	1	1	0	0	1.4	0.5	2.8
PF02129	X-Pro dipeptidyl-peptidase (S15 family)	2	4	3	1	7	3	1	1	0	3.4	1.25	2.7

The table shows the number of predicted proteins that contain each Pfam motif.

Key: B. cin = *Botrytis cinerea*, G. zea = *Gibberella zeae*, M. gri = *M. grisea*, S. scl = *Sclerotinia sclerotiorum*, S. nod = *Stagonospora nodorum*, A. nid = *Aspergillus nidulans*, C.glo = *Chaetomium globosum*, N. cra = *Neurospora crassa*, T. ree = *Trichoderma reesei*

1 Mean number of predicted proteins in pathogen proteomes.

2 Mean number of predicted proteins in non-pathogen proteomes.

3 path/non-path

### Comparative secretome analysis of phytopathogenic and saprotrophic filamentous ascomycetes

**Figure 2:**
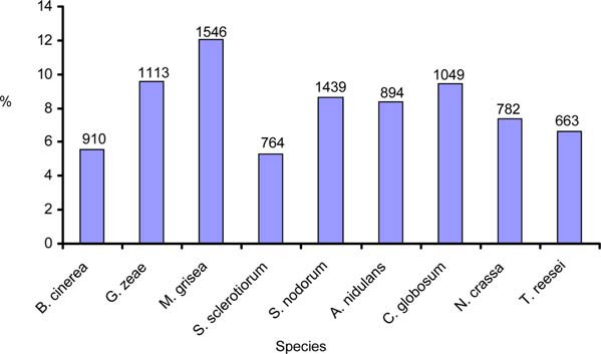
Bar chart showing the percentage of the total proteome that is predicted to be secreted in each fungal species. The number of secreted proteins is indicated at the top of each bar.

Studies in bacterial pathogens and oomycetes have shown that a range of secreted proteins known as effectors are important for establishing infection of the host plant [73], [74]. These secreted proteins may disable plant defences and subvert cellular processes to suit the needs of invading pathogens. Therefore, we decided also to compare gene family size in the secretomes of phytopathogens and non-pathogens. There are a number of programs available that predict whether a protein is likely to be secreted, although the predictions they give significantly differ from each other. Therefore we defined the secretome of each fungal species based on those proteins that are predicted to be secreted by two different programs: SignalP 3.0 [75] and WoLFPSORT [76]. The size of each secretome is summarised in Figure 2. Even when using two programs, the sizes of predicted secretomes can vary greatly. For example, a similar analysis for *M. grisea* using SignalP and ProtComp (www.Softberry.com) predicted only 739 secreted proteins (out of a proteome of 11,109) compared to our prediction of 1,546 secreted proteins (out of a proteome of 12,841) [22]. The size of the secretomes for each species varied from 5%–12% of the total proteome. Overall, the size of the secretomes from phytopathogens did not differ greatly from that of non-pathogens.

Table 8 shows a list of Pfam motifs, not found in the secretomes of non-pathogenic filamentous ascomycetes, that were present in at least three phytopathogenic fungal species. The *Isochorismatase* motif (PF00857) was found in the secretomes of all five species of phytopathogen. Isochorismatase catalyses the conversion of isochorismate to 2,3-dihydroxybenzoate and pyruvate. It has been implicated in the synthesis of the anti-microbial compound phenazine by *Pseudomonas aeruginosa* [77] and the siderophore, enterobactin, by *Escherichia coli* [78]. The isochorismatase motif is also found in a number of hydrolases, such as nicotinamidase that converts nicotinamide to nicotinic acid [79]. Members of this family are found in all filamentous ascomycetes, but interestingly they are only secreted in phytopathogens. Salicylic acid is synthesised in plants in response to pathogen attack and mediates plant defences. As isochorismate is a precursor of salicyclic acid [80], it may be worth speculating that isochorismatases secreted by fungi could act to reduce salicylic acid accumulation in response to pathogen attack and thus inhibit plant defence responses. The secreted isochorismatases (apart from one of the proteins from *S. nodorum*) all show sequence similarity to ycaC from *E. coli*, an octameric hydrolase of unknown function [81]. Pfam motifs found in the secretomes of at least three species of phytopathogens, but not in any of the non-pathogens also include those found in enzymes potentially involved in detoxification, such as arylesterase and amidohydrolase, and also beta-ketoacyl synthase, which catalyses the condensation of malonyl-ACP with a growing fatty acid chain and is found as a component of a number of enzyme systems, including fatty acid synthases and polyketide synthases [82], [83].

**Table 8:** Pfam motifs that are found in the secretomes from at least three species of phytopathogen but in no species of filamentous ascomycete non-pathogen.

Pfam accession	Pfam description	*B. cinerea*	*G. zeae*	*M. grisea*	*S. sclerotiorum*	*S. nodorum*
PF00857	Isochorismatase family	1	1	1	1	2
PF01731	Arylesterase	1	2	0	1	1
PF04113	Gpi16 subunit, GPI transamidase component	1	1	1	1	0
PF07969	Amidohydrolase family	1	2	0	1	1
PF00109	Beta-ketoacyl synthase, N-terminal domain	1	0	1	1	0
PF01156	Inosine-uridine preferring nucleoside hydrolase	1	0	1	1	0
PF02801	Beta-ketoacyl synthase, C-terminal domain	1	0	1	1	0
PF03134	TB2/DP1, HVA22 family	0	0	1	1	1
PF04253	Transferrin receptor-like dimerisation domain	0	2	1	0	1
PF05390	Yeast cell wall synthesis protein KRE9/KNH1	1	0	0	1	1

Table shows the number of predicted proteins that contain each Pfam motif.

Table 9 shows a list of Pfam motifs that are more common in the secretomes of phytopathogens as compared to saprotrophs. These include a number of secreted proteases, transcription factors and components of signal transduction pathways. The *Kelch* domain (PF01344) shows the most striking difference in distribution between phytopathogenic and non-pathogenic genomes. This 50-residue domain is found in a number of actin-binding proteins [84], as well as enzymes such as galactose oxidase and neuraminidase. The putative function of each secreted Kelch domain-containing protein was ascertained by performing a BLAST search against the NCBI non-redundant protein database (Table 10). A number of these seem to be galactose oxidases, enzymes which catalyse the oxidation of a range of primary alcohols, including galactose, to the corresponding aldehyde with the concomitant reduction of oxygen to hydrogen peroxide (H_2_O_2_) [85]. Galactose oxidase shares a copper radical oxidase motif with the hydrogen peroxide-generating glyoxal oxidases involved in lignin-degradation in *Phanerochaete chrysosporium* [86]. H_2_O_2_-producing copper oxidases have been shown to have roles in morphogenesis, in the corn-smut fungus *Ustilago maydis* for example, a glyoxal oxidase is required for filamentous growth and pathogenicity [87] and a galactose oxidase is involved in fruiting body formation in the gram-negative bacterium *Stigmatella aurantiaca* [88]. Interestingly, the list of Pfam motifs more common in the secretomes of phytopathogens also includes those found in copper amine oxidases, H_2_O_2_-generating enzymes that catalyse the oxidative deamination of primary amines to the corresponding aldehydes [89] and peroxidases, haem-containing enzymes that use hydrogen peroxide as the electron acceptor to catalyse a number of oxidative reactions. Secreted fungal peroxidases include enzymes involved in lignin breakdown by the white rot fungus *Phanerochaete chrysosporium* [90], but in plants they generate reactive oxygen species and are involved in defence responses and growth induction [91]. A number of other secreted Kelch domain-containing proteins have similarity to proteins of unknown function from species of the bacterial phytopathogen *Xanthomonas*. Many Kelch domain-containing proteins are involved in cytoskeletal rearrangement and cell morphology [92], [93]. It may be worth speculating that secreted Kelch domain-containing proteins could act as effectors, causing changes in the arrangement of the cytoskeleton of infected plants to aid the proliferation of fungal hyphae. It has recently been shown, for example, that *M. grisea* co-opts plasmodesmata to move from cell to cell in infected rice leaves [94] and would therefore need to peturb cytoskeletal organisation in rice epidermal cells. There are other Pfam domains that are more common in the secretomes of phytopathogens that may potentially be found in effectors such as the *PAN* domain (PF00024), that mediates protein-protein and protein-carbohydrate interactions [95] and the *F5/8 type C* domain (PF00754), found in the discoidin family of proteins involved in cell-adhesion or developmental processes [96].

**Table 9:** Pfam motifs that are at least twice as common in the secretomes of filamentous ascomycete phytopathogens as compared to filamentous ascomycete non-pathogens.

Accession	Pfam description	B. cin	G. zea	M. gri	S. scl	S. nod	A. nid	C. glo	N. cra	T. ree	path^1^	non-path^2^	Ratio^3^
PF01344	Kelch motif	2	3	4	1	5	0	1	0	0	3	0.25	12.0
PF00024	PAN domain	2	9	0	1	3	0	0	0	2	3	0.5	6.0
PF04082	Fungal specific transcription factor domain	2	2	0	1	1	1	0	0	0	1.2	0.25	4.8
PF00089	Trypsin	1	2	3	1	3	1	0	0	1	2	0.5	4.0
PF00232	Glycosyl hydrolase family 1	0	1	1	2	1	1	0	0	0	1	0.25	4.0
PF01019	Gamma-glutamyltranspeptidase	1	1	1	1	1	1	0	0	0	1	0.25	4.0
PF01161	Phosphatidylethanolamine-binding protein	1	2	4	0	3	1	1	0	0	2	0.5	4.0
PF02128	Fungalysin metallopeptidase (M36)	0	1	2	0	2	0	0	0	1	1	0.25	4.0
PF03403	Platelet-activating factor acetylhydrolase, plasma/intracellular isoform II	1	0	1	1	2	0	1	0	0	1	0.25	4.0
PF04909	Amidohydrolase	1	1	0	1	2	0	0	1	0	1	0.25	4.0
PF07504	Fungalysin/Thermolysin Propeptide Motif	0	1	2	0	2	0	0	0	1	1	0.25	4.0
PF08244	Glycosyl hydrolases family 32 C terminal	0	1	1	1	2	0	0	1	0	1	0.25	4.0
PF00246	Zinc carboxypeptidase	2	7	9	1	8	1	1	2	2	5.4	1.5	3.6
PF00445	Ribonuclease T2 family	2	3	1	2	1	1	0	1	0	1.8	0.5	3.6
PF03572	Peptidase family S41	1	0	3	0	5	0	0	1	1	1.8	0.5	3.6
PF07883	Cupin domain	2	2	1	2	2	1	0	0	1	1.8	0.5	3.6
PF01083	Cutinase	9	10	12	7	9	2	5	2	2	9.4	2.75	3.4
PF00710	Asparaginase	1	1	0	0	2	1	0	0	0	0.8	0.25	3.2
PF00753	Metallo-beta-lactamase superfamily	2	1	1	0	0	0	0	0	1	0.8	0.25	3.2
PF00754	F5/8 type C domain	1	2	0	0	1	0	0	1	0	0.8	0.25	3.2
PF01179	Copper amine oxidase, enzyme domain	0	3	2	0	3	1	0	1	0	1.6	0.5	3.2
PF01679	Uncharacterized protein family UPF0057	0	1	2	0	1	1	0	0	0	0.8	0.25	3.2
PF02244	Carboxypeptidase activation peptide	1	0	1	1	1	0	0	0	1	0.8	0.25	3.2
PF03694	Erg28 like protein	1	0	1	1	1	1	0	0	0	0.8	0.25	3.2
PF00141	Peroxidase	1	3	4	1	5	0	1	1	2	2.8	1	2.8
PF00194	Eukaryotic-type carbonic anhydrase	1	2	2	1	1	0	1	0	1	1.4	0.5	2.8
PF00187	Chitin recognition protein	4	7	17	5	14	6	7	0	1	9.4	3.5	2.7
PF00295	Glycosyl hydrolases family 28	17	6	3	17	4	9	1	2	3	9.4	3.75	2.5

Table shows the number of predicted proteins that contain each Pfam motif.

Key: B. cin = *Botrytis cinerea*, G. zea = *Gibberella zeae*, M. gri = *M. grisea*, S. scl = *Sclerotinia sclerotiorum*, S. nod = *Stagonospora nodorum*, A. nid = *Aspergillus nidulans*, C.glo = *Chaetomium globosum*, N. cra = *Neurospora crassa*, T. ree = *Trichoderma reesei*

1 Mean number of predicted proteins in pathogen secretomess.

2 Mean number of predicted proteins in non-pathogen secretomes.

3 path/non-path

**Table 10:** Secreted Kelch-domain containing proteins

Gene locus	Species	Top non-hypothetical hit vs NCBI non-redundant protein database^1^
BC1G_02702.1	*Botrytis cinerea*	ring canal kelch-like protein (*Xanthomonas campestris pv. campestris*) (AAM43333.1) (8e-30)
BC1G_12145.1	*Botrytis cinerea*	galactose oxidase (*Gibberella zeae*) (XP_391208.1) (1e-160)
FG00251.1	*Gibberella zeae*	galactose oxidase (*Gibberella zeae*) (XP_391208.1) (0)
FG09093.1	*Gibberella zeae*	galactose oxidase (*Cladobotryum dendroides*) (A38084) (1e-126)
FG09142.1	*Gibberella zeae*	ring canal kelch-like protein (*Xanthomonas campestris pv. campestris*) (AAM43333.1) (5e-32)
MGG_02368.5	*Magnaporthe grisea*	galactose oxidase (*Cladobotryum dendroides*) (A38084) (1e-117)
MGG_03826.5	*Magnaporthe grisea*	ring canal kelch-like protein (*Xanthomonas campestris pv. campestris*) (AAM43333.1) (7e-22)
MGG_04086.5	*Magnaporthe grisea*	ring canal kelch-like protein (*Xanthomonas campestris pv. campestris*) (AAM43333.1) (9e-24)
MGG_10013.5	*Magnaporthe grisea*	ring canal kelch-like protein (*Xanthomonas campestris pv. campestris*) (AAM43333.1) (9e-34)
SS1G_03276.1	*Sclerotinia sclerotiorum*	beta-scruin (*Limulus polyphemus*) (Q25386) (1e-07)
SNU05548.1	*Stagonospora nodorum*	Kelch repeat (*Herpetosiphon aurantiacus*) (ZP_01426654) (2e-18)
SNU06096.1	*Stagonospora nodorum*	ring canal kelch-like protein (*Xanthomonas axonopodis pv. citri*) (NP_644535.1) (9e-30)
SNU08346.1	*Stagonospora nodorum*	epithiospecifier (*Arabidopsis thaliana*) (AAL14622.1) (3e-11)
SNU11576.1	*Stagonospora nodorum*	galactose oxidase (*Gibberella zeae*) (XP_391208.1) (1e-120)
SNU15302.1	*Stagonospora nodorum*	galactose oxidase (*Gibberella zeae*) (XP_391208.1) (0)
CHG08026.1	*Chaetomium globosum*	Kelch (*Herpetosiphon aurantiacus*) (ZP_01423335.1) (1e-09)

1 Species, accession number and E-value of BLAST search (using BLASTP) shown in brackets in that order.

## Discussion

One of the most fundamental aims in plant pathology research is to define precisely the difference between pathogenic and non-pathogenic microorganisms. The answer cannot be one of simple phylogeny, because phytopathogenic species are found in all taxonomic divisions of fungi and are often closely related to non-pathogenic species [3]. Before the availability of genomic sequences and high throughput approaches to study gene function [20], research was concentrated on the search for single pathogenicity factors; genes that are dispensable for saprophytic growth but essential for successful infection of the host plant [4], [97]. However, rather than encoding novel proteins found only in phytopathogens, the majority of pathogenicity factors discovered in this way have been found to be involved in signalling cascades and metabolic pathways and hence are conserved in most species of fungi [5]. Components of signalling cascades that in the budding yeast *S. cerevisiae* are responsible for responses to pheromones, nutritional starvation and osmotic stress [9] have in many cases evolved different roles in the life cycle of pathogens, such as controlling appressorium formation, dimorphism and growth [10]. Although the central components of signalling are conserved between phytopathogens and *S. cerevisiae*, the receptors are often different, reflecting the different environmental cues to which the pathogen needs to respond [11], [98].

Analysis of all available genome sequences from a wider range of fungal species has for the first time allowed us to address the differences between phytopathogens and non-pathogens at a whole genome level. For this purpose, the *e-Fungi* data warehouse provides a means to interrogate the vast amounts of genomic and functional data available in a simple integrated manner [26]. Previous research, in which EST datasets were compared with genomic sequences, suggested that the expressed gene inventories of phytopathogenic species were not significantly more similar to one another than to those of saprotrophic filamentous fungi [99]. We clustered sets of predicted proteins from 36 different species of fungi and oomycetes into groups of potential orthologues and the species distribution of members of each cluster was ascertained. There were no clusters that were completely specific to phytopathogenic species across both fungi and oomycetes, suggesting that the presence of novel, universal pathogenicity factors in the genomes of phytopathogens is unlikely. This was confirmed by looking at clusters containing empirically defined pathogenicity factors, where homologues of many of these were found in all species studied and none were conserved in the genomes only of phytopathogens. A small number were only found in a single species of fungus and probably represented proteins that are highly specialised for a particular role in a specific pathogenic species, for example in host-plant recognition [54]. Previous research also suggested that the gene inventories of filamentous fungi were more similar to each other than to those of unicellular yeasts [99]. Analysis of the clusters of similar proteins show some clusters that are found in all species of filamentous fungi (including ascomycetes, basidiomycetes and zygomycetes) but are not present in the genomes of yeasts, consistent with the original conclusion. These contain a number of proteins that are likely to be involved in morphological changes associated with the more complex filamentous lifestyle, as well those involved in secondary metabolism and signalling cascades that are not found in yeasts. In particular, our results suggest that filamentous fungi use a wider variety of lipid molecules for the purpose of signalling. Some of these may act as pheromones, or hormones– chemical messengers diffusing from one cell to another to elicit a physiological or developmental response [37]. A number of these innovations to the filamentous lifestyle may serve important roles in pathogenesis as well, because homologues of a number of pathogenicity factors are found only in filamentous ascomycetes. The distribution of filamentous fungi-specific proteins, such as involved in those cytoskeletal rearrangements and fruiting body formation, throughout the fungal kingdom (and in some cases in oomycetes as well), suggests that the last common ancestral fungus may well have been multi-cellular and the evolution of uni-cellular fungi was likely associated with massive gene loss. For example, it has been shown that early in ascomycete evolution there was a proliferation of subtilase-type protease-encoding genes that have been retained in some filamentous ascomycete lineages, but lost in the yeast lineage [100].

It has previously been speculated that the evolution of phytopathogenesis was associated with the expansion of certain gene families [1]. Duplication of an ancestral gene, followed by mutation allows members of the family to take on new functions [101]. For example, genomes of the filamentous ascomycetes studied here have between 40 and 140 cytochrome P450-encoding genes (data not shown) that are involved in toxin biosynthesis, lipid metabolism, alkane assimilation and detoxification [102] and which probably arose via gene duplication and functional diversification. In contrast, the genome of the budding yeast *S. cerevisiae* has only three cytochrome P450-encoding enzymes. We have shown here that there are likely to be large differences in the gene inventories of filamentous fungi compared to unicellular yeasts.

To study the differences between phytopathogenic and saprophytic fungi, we concentrated on the filamentous ascomycetes where there are a number of phytopathogenic species genomes have been sequenced along with closely related non-pathogens. Protein families were defined using Pfam motifs [57] and the predicted protein sets for each species analysed in order to identify domains that were specific to or more common in the genomes of phytopathogens. Not surprisingly, many of the protein families we identified are likely to be associated with pathogenic processes such as plant cell wall degradation, toxin biosynthesis, formation of reactive oxygen species and detoxification [5]. Studies of bacterial phytopathogens have shown the importance of effectors, secreted proteins that disable plant defences and subvert metabolic and morphological processes for the benefit of the invading pathogen and which require delivery via a type III secretion system that are often deployed during pathogenesis [73]. Bacterial type III secreted effectors (T3SEs) have been shown to target salicyclic acid and abscisic acid-dependent defences, host vesicle trafficking, transcription and RNA metabolism, and several components of the plant defence signalling networks [103]. Very recently, potential effector-encoding genes have been identified in the genomes of several species of oomycete pathogens and are defined by the presence of a conserved RXLR-EER motif downstream of the signal peptide sequence [74]. The RXLR-EER motif is necessary for delivery of effector proteins into host plant cells and is therefore critical to their biological activity [74].

To identify potential fungal effectors, we compared Pfam motif frequency between the secretomes of phytopathogens and non-pathogens. This analysis identified potential effector-encoding genes, including secreted proteases, transcription factors and proteins that may be involved in cytoskeletal rearrangements (such as Kelch-domain containing proteins) and protein-protein interactions, as well as a group of pathogen-specific secreted isochorisimatases that potentially could suppress salicyclic acid-dependent host plant defences. Bacterial T3SEs are injected directly into the host cytoplasm via the type III secretion injection apparatus [73]. In contrast, the potential fungal effectors identified in this study appear to be secreted by the normal cellular secretory pathway via the endoplasmic reticulum and the mechanism by which fungal effectors might be taken up by plant cells and enter into the host cytoplasm is currently unknown.

Although the evolution of phytopathogenicity is likely to have happened several times and the lifestyles of these fungi are diverse, a comparison of gene inventories of a number of species using a powerful resource, such as *e-Fungi*, has allowed us to pinpoint new gene families that may serve important roles in the virulence of phytopathogens, allowing their selection for gene functional studies, that are currently in progress. The analyses deployed here may also offer a blueprint for the types of larger, more comprehensive studies that will be necessary to interpret the large flow of genetic data that will result from next generation DNA sequence analysis utilizing both a much wider variety of fungal pathogen species and also large sets of individual isolates of existing species.

## Materials and Methods

### Clustering of sequences

Sets of predicted proteins were downloaded for each of the 36 genomes from respective sequencing project websites (Table 1). Proteins less than 40 amino acids in length were not included in this analysis. Proteins were clustered using “all against all” BLASTP [104] followed by Markov Chain Clustering (MCL) [27] with 2.5 as a moderate inflation value and 10^−10^ as an E-value cut-off, as described previously [28], [29]. Clusters were annotated based on best hit against Swiss-Prot protein database [105] of members of that cluster (e-value <10^−20^ using BLASTP), or Pfam motifs contained in proteins from the cluster in the absence of Swiss-Prot hits.

### Identification of Pfam motifs

The Pfam-A library from release 18.0 of the Pfam database was downloaded from the Pfam website (http://www.sanger.ac.uk/Software/Pfam/). This library contains 7973 protein models constructed from manually curated multiple alignments and covers 75% of proteins in UniProt [44], [57]. This library was used to analyse the sequences of predicted proteins for all 36 fungal genomes to identify the Pfam motifs that each protein contains. The analysis was performed using the “pfam_scan” perl script (version 0.5) downloaded from the Pfam website and HMMER software (downloaded from http://hmmer.wustl.edu/). Default thresholds were used, which are hand-curated for every family and designed to minimise false positives [44].

### Identification of secreted proteins

The N-terminal sequence of each predicted protein from the 36 fungal genomes used in this study was analysed for the presence of a signal peptide using SignalP 3.0 [75] and sub-cellular localisation was predicted using WoLF PSORT [76]. Both these programs were installed locally. SignalP 3.0 uses two different algorithms to identify signal sequences. The secretome for each fungal species was defined as containing those proteins that were predicted have a signal peptide by both prediction algorithms from SignalP 3.0 and also predicted to be extracellular by WoLF PSORT.

### Data analysis

All the data produced, as described above, was stored in the *e-Fungi* data warehouse [26] from which it can be accessed via a web-interface (http://www.e-fungi.org.uk/). Analyses described in this study were performed using the *e-Fungi* database.

## Supporting Information

Table S1 (0.08 MB XLS)

Table S2 (0.02 MB XLS)

Table S3 (0.16 MB XLS)

Table S4 (0.06 MB XLS)

Table S5 (0.03 MB XLS)

Table S6 (0.04 MB XLS)
