# Supplementary material for: Comparative Genome Analysis of Filamentous Fungi Reveals Gene Family Expansions Associated with Fungal Pathogenesis
Source: PLoS One. 2008 Jun 4;3(6):e2300. doi: 10.1371/journal.pone.0002300 (PMC2409186; doi:10.1371/journal.pone.0002300)
Supplement: Table S6 — (0.04 MB XLS) [file pone.0002300.s006.doc]

**Table S6**

| *Species* | *genome coverage* | *genome size (Mb)* | *number of predicted proteins* | *number of predicted proteins / Mb genome* |
| --- | --- | --- | --- | --- |
| *Aspergillus nidulans* | 13 x | 30.06 | 10,701 | 356 |
| *Botrytis cinerea* | 5.41 x | 42.663 | 16,448 | 386 |
| *Chaetomium globosum* | 7 x | 34.887 | 11,124 | 319 |
| *Fusarium graminearum* | 10 x | 36.43 | 11,640 | 320 |
| *Magnaporthe grisea* | 7 x | 41.624 | 12,841 | 308 |
| *Neurospora crassa* | 10 x | 39 | 10,620 | 272 |
| *Sclerotinia sclerotiorum* | 8 x | 38.33 | 14,522 | 379 |
| *Stagonospora nodorum* | 10 x | 37.236 | 16,597 | 446 |
| *Trichoderma reesei* | data not available | 33 | 9,997 | 303 |
